# EXPLORING SELF-COMPASSION AND EXPANDED OUTCOMES: A THEORETICAL MODEL OF CAREGIVING

**DOI:** 10.1093/geroni/igae098.4183

**Published:** 2024-12-31

**Authors:** Claire Grant, Katherine Judge

**Affiliations:** Cleveland State University, Cleveland, Ohio, United States; Cleveland State University, Cleveland, Ohio, United States

## Abstract

Expanding the theoretical understanding of the caregiver stress process is integral to the development and tailoring of evidence-based interventions for caregivers of people living with dementia (PLWD). Recent calls within the literature have suggested testing new theoretical models to expand the investigation of positive predictors and mechanisms of action to a broader range of outcomes including positive aspects of caregiving. The proposed poster will address both gaps within the literature by testing a model of self-compassion developed by Cha and colleagues (2023) adapted for caregivers of PLWDs. Self-compassion is a construct relating to positive self-concept including key tenets of self-kindness, common humanity, and mindfulness. It has been identified as a strong predictor of various caregiver wellbeing outcomes and is increasingly used in intervention research. The theoretical model investigates self-compassion as a main predictor and proposes two parallel lines of mediation (general resources allowing for self-control behavior and specific psychological resources including coping and emotion regulation strategies) to impact a range of outcomes including depression, anxiety, and perceived stress, along with more novel positive outcomes including social support, and positive aspects of caregiving. Testing the model in a sample of caregivers of PLWDs will provide important insights into predictors of both positive and negative outcomes with important implications in development of evidence-based interventions as potential mechanisms of change are investigated. The poster will present a review of the literature supporting the model and discuss ongoing data collection with analysis plans. Further implications across caregiver theory and intervention development will be discussed.

